# Risk-based efficacy of systemic and local antibiotics in nonsurgical treatment of peri-implantitis: A systematic review and meta-analysis

**DOI:** 10.34172/japid.026.3971

**Published:** 2025-12-09

**Authors:** Masoumeh Faramarzi, Sajjad Pourkaveh, Adileh Shirmohammadi, Mehrnoosh Sadighi, Sevda Mashhadi Jolfaei, Elnaz Ziaeirad, Amir Zandesh

**Affiliations:** ^1^Department of Periodontics, Faculty of Dentistry, Tabriz University of Medical Sciences, Tabriz, Iran; ^2^Student Research Committee, Tabriz University of Medical Sciences, Tabriz, Iran

**Keywords:** Antibiotics, Meta-analysis, Peri-implantitis, Systematic review

## Abstract

**Background.:**

Peri-implantitis is a prevalent inflammatory condition that compromises the long-term stability of dental implants. With its rising incidence, adjunctive therapies to nonsurgical mechanical debridement, particularly antibiotics, have gained attention. This systematic review evaluated the efficacy of systemic and local antibiotics as adjuncts to nonsurgical peri-implantitis treatment, focusing on probing depth (PD) reduction, bleeding on probing (BOP), and clinical attachment level (CAL).

**Methods.:**

In accordance with the PRISMA 2020 guidelines, randomized controlled trials (RCTs) were screened. Data extraction and quality assessment were performed independently by two reviewers, and the quantitative synthesis was conducted using the CMA3 software.

**Results.:**

Out of 93 screened records, six RCTs were included in this systematic review. Five studies investigated the effects of systemic treatment, and only one RCT reported the outcomes of topical antibiotic use. The quality of the studies was unclear in five studies and low in one study. Both systemic and local antibiotics demonstrated clinical benefits when combined with mechanical debridement. Reported adverse effects were generally mild. Overall, antibiotic therapy was associated with a greater decrease in PD (mean difference: -0.66, 95% confidence intervals: -1.014, -0.318, *P*<0.001; I^2^ : 76.97, *P* value for heterogeneity<0.01). In the subgroup analyses, systemic therapy was associated with better outcomes.

**Conclusion.:**

Based on the limited available evidence, antibiotics may offer adjunctive benefits in the nonsurgical management of peri-implantitis. Protocol heterogeneity and limited long-term data highlight the need for standardized regimens, assessment of antimicrobial resistance, high-quality long-term RCTs, and cost-effective studies in this regard.

## Graphical Abstract

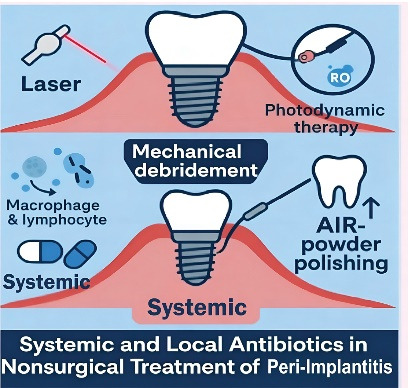

## Introduction

 Peri-implantitis is characterized by inflammation in the tissues surrounding dental implants, often leading to the loss of supporting bone and, if untreated, implant failure.^1,2^This condition presents a significant challenge in implant dentistry due to its prevalence, the complex interplay of contributing factors, and the difficulty in achieving consistent treatment outcomes.^3^ The exponential increase in the placement of dental implants worldwide has been accompanied by a parallel rise in the incidence of peri-implant diseases.^4-6^ Peri-implantitis and peri-implant mucositis fall under the category of peri-implant conditions.^7,8^Peri-implantitis is a disease associated with bone loss, osseointegration disability, increased pocket formation, and affects 19.53% of patients and 12.53% of implant locations.^9^ Peri-implant mucositis is a temporary process of inflammation, primarily affecting 46.83% of individuals and 29.48% of implants. Biofilm or dental plaque is the most important etiological factor.^10,11^ Peri-implantitis has comparable causal and pathological elements with periodontal disease.^12,13^ Many pathogenic organisms, including *Tannerella forsythia* and *Aggregatibacter actinomycetemcomitans,* have been isolated in both conditions.^14,15^

 A history of periodontitis is consistently identified as the single most significant risk factor for peri-implantitis,^15^ as both conditions share a similar infectious etiology involving bacterial biofilm and a dysregulated host immune response.^16^ Smoking is another confirmed risk factor with moderate certainty evidence. The 2024 Academy of Osseointegration/American Academy of Periodontology (AO/AAP) consensus identifies smoking as a key behavioral risk factor.^17-19^ Poorly-controlled diabetes mellitus is also strongly associated with a higher risk of peri-implantitis.^17^Poorly controlled blood glucose levels can compromise osseointegration and lead to greater marginal bone loss, directly affecting implant health.^16^The 2024 AO/AAP consensus explicitly links metabolic health to the risk of peri-implantitis^20^ and recommends a comprehensive assessment of factors such as body mass index (BMI) and glucose levels to guide a multifaceted treatment approach.^21^ Poor plaque control is also considered the principal etiological factor in peri-implant disease. The absence of regular supportive maintenance therapy is a critical behavioral risk factor for the initiation and progression of the disease.^22^

 Nonsurgical approaches to peri-implantitis have been brought to the limelight due to their non-invasive procedure^23^; however, mechanical debridement, a fundamental component of all nonsurgical therapy, is often insufficient as monotherapy, particularly in advanced cases.^24^ During the process, mechanical debridement and the detoxification of the implant surface are combined with laser therapy, air-polishing, and antimicrobial agents.^25-27^ Systemic antibiotics are the most widely used, but side effects and risk of antibiotic resistance_,_^28^ lead to a trend towards topical antibiotics.^29,30^ Systemic antibiotics, such as amoxicillin and metronidazole, provide a significantly greater reduction in probing depth (PD) and can lead to radiographic bone gain, especially in advanced cases characterized by deep pockets (PD > 6 mm)^31^ and increased mucosal recession and improved bleeding on probing (BOP).^32^ In contrast, local/topical antibiotics offer a modest yet valuable adjunctive benefit, representing a suitable alternative for patients who are at risk of systemic complications or for those with less severe disease.^24^

 Park et al^33^ reported that metronidazole‒minocycline ointment as an adjunct treatment, along with debridement, is more effective than debridement alone.^33^ Polymeri et al shed light on concerns about the sustainability of systemic antibiotic regimens, given factors such as antibiotic resistance.^34^ Numerous systematic reviews and meta-analyses have investigated the efficacy of antibiotics for treating peri-implantitis. Grusovin et al^29^ reported that local antibiotics have a significant impact on PD and BOP, compared with mechanical debridement alone. Likewise, Toledano et al proved that local antibiotic therapy is efficient in inflamed tissue regression and decreases bacterial counts.^25^ In the current systematic review, the primary objective was to evaluate the efficacy of topical and systemic antibiotics as adjuncts to nonsurgical management of peri-implantitis by analyzing clinical data from randomized controlled trials (RCTs). By synthesizing recent evidence, this review aims to provide a clear, risk-based framework for clinicians to make informed decisions about personalized treatment protocols.

## Methods

 This systematic review was conducted in accordance with the PRISMA 2020 guidelines.^35^

###  Search strategy

 A comprehensive electronic literature search was performed across multiple databases, including PubMed/MEDLINE, Embase, and the Cochrane Central Register of Controlled Trials (CENTRAL). The search strategy was designed to identify all relevant studies published from January 2018 to August 2025. This timeframe was strategically chosen to focus on contemporary evidence, reflecting recent advancements in implant materials, diagnostic criteria, and therapeutic protocols, while excluding older studies with potentially confounding methodologies. The search used a combination of MeSH terms and keywords, including “peri-implantitis,” “dental implants,” “nonsurgical therapy,” “anti-bacterial agents,” “systemic antibiotics,” and “local antibiotics.” Additionally, the reference lists of included articles and relevant systematic reviews were manually screened to identify any further eligible studies.

###  Eligibility criteria

 Studies were selected based on the following inclusion criteria: RCTs evaluating the adjunctive use of systemic or local antibiotics in the nonsurgical treatment of peri-implantitis. Exclusion criteria were: observational studies, case reports, reviews, opinion articles, and studies with incomplete data.

###  Study selection

 The study selection process was carried out in two phases. Initially, the titles and abstracts of the identified studies were reviewed to assess eligibility. In the subsequent phase, a thorough examination of the full-text articles was conducted to determine inclusion based on the defined criteria. Two independent reviewers evaluated each study, and disagreements were resolved through discussion or by a third reviewer.

###  Data extraction and quality assessment

 Data were extracted from the included studies using a standardized extraction form. Information collected included study characteristics (authors, year of publication, study design), sample size and patient demographics, type of antibiotic (topical or systemic) and treatment protocol, and outcomes (decrease in PD, BOP, clinical attachment level [CAL]). The quality of included randomized controlled trials was assessed using the Cochrane Risk of Bias Tool.

###  Data synthesis

 A qualitative approach was adopted to describe the clinical outcomes.^36^ In addition, means and standard deviations for changes in PD in the antibiotic and control groups were used for quantitative synthesis. Meta-analysis was conducted using the third version of Comprehensive Meta-Analysis (CMA3) with 95% confidence intervals and an 0.05 level of significance for *P *value. Heterogeneity was assessed using the I^2^ statistic; given significant heterogeneity, a random-effect analysis was conducted, and the results were finally presented in a forest plot.

## Results

###  Study selection and characteristics

**Figure 1:**
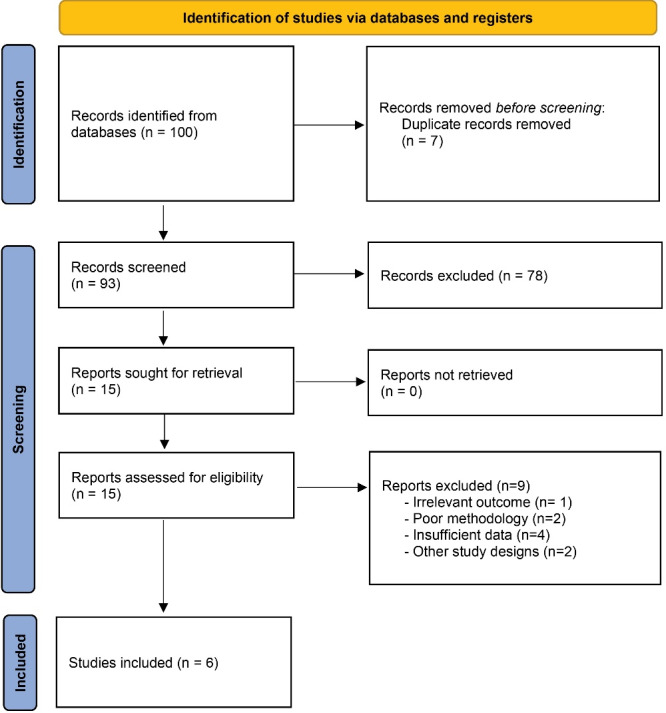
PRISMA 2020 flow diagram.From:Page et al.^35^ For more information, visit: http://www.prisma-statement.org/

 The process began with the identification of 100 records from various databases. After removing 7 duplicates, 93 records remained for screening. During the screening phase, these records were thoroughly evaluated, and 80 were excluded based on predefined criteria. Fifteen reports were then assessed for eligibility, leading to the exclusion of 9 reports, and finally, 6 RCTs were included (Figure 1).^33,34,37-40^ The studies included in this review were conducted between 2019 and 2022. Table 1 presents the details of the included studies. Ahmed et al’s^39^ study did not report the conflicts of interest, and there were no conflicts of interest in other studies, except for De Waal et al’s^40^ study.

###  Narrative synthesis of the outcomes

Table 2 presents the details of the outcomes of the included studies. Regarding PD reduction, primary evidence from some included RCTs demonstrated notable reductions in PD. For instance, the multicenter trial by Park et al,^33^ which assessed a topical metronidazole-minocycline ointment, reported a statistically significant mean PD reduction in the test group compared with the control group receiving mechanical debridement alone. In Blanco et al’s^37^ study, metronidazole was associated with greater improvements in PD. The clinical trial by Polymeri et al,^34^ evaluating systemic amoxicillin and metronidazole, found a significant decrease in the proportion of deep pockets; however, the adjunctive use of systemic amoxicillin and metronidazole did not show significantly better results compared to the nonsurgical treatment alone, which was evident in Shibli et al’s^38^ and De Waal et al’s^40^ studies, too. Regarding the BOP, in Polymeri et al’s,^34^ Shibli et al’s,^38^ and Park et al’s^33^ studies, antibiotic therapy was not associated with significant improvements. CAL did not improve significantly in Shibli et al’s^38^ and De Waal et al’s^40^ studies.

 BOP: bleeding on probing; PD: probing depth; CAL: clinical attachment level

 Regarding microbiological changes, evidence indicates beneficial shifts in the peri-implant microbiome. The RCT by Park et al^33^ used microbiological analysis and found a significant reduction in the counts of key peri-implant pathogens, such as *Porphyromonas gingivalis*, in the local antibiotic group; however, in Shibli et al’s^38^ study, red complex pathogens did not differ significantly. Regarding adverse events, the included RCTs reported a low incidence. In studies by Polymeri et al^34^ and Park et al,^33^ no side effects were reported. In Blanco et al’s^37^ study, adverse events (including gastrointestinal disorders, headache, metallic taste, and oral tissue alterations) were comparable between the groups. In De Waal et al’s^40^ study, no significant difference was observed in this regard.

###  Meta-analysis 

**Figure 2:**
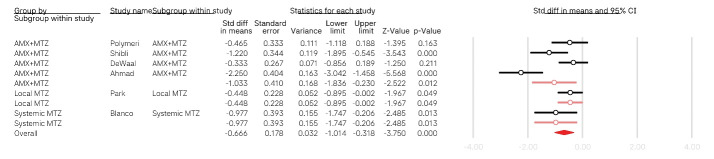
The results of the meta-analysis

 Based on the conducted meta-analysis (Figure 2), antibiotic therapy was associated with a greater decrease in PD (mean difference: -0.66, 95% confidence intervals: -1.014, -0.318, *P* < 0.001; I^2^: 76.97, *P* value for heterogeneity < 0.01). In the subgroup analyses, systemic therapy was associated with better outcomes. A combination of amoxicillin and metronidazole was the most effective intervention for decreasing PD (mean difference: -1.033, 95% confidence intervals: -1.836, -0.230, *P* = 0.01; I^2^: 83.53, *P* value for heterogeneity < 0.01).

**Table 1:** Study characteristics of the included studies

**Study**	**Country**	**Sample size**	**Age/Male ratio **	**Diagnostic criteria**	**Antibiotics and details**	**Outcome measures**	**Study quality**
Polymeri et al (2022)^34^	Netherlands	37	60.8 ± 14.8/68%; 58.3 ± 13.9/61%	PD ≥ 5 mm, BOP, marginal bone loss ≥ 3 mm	Systemic amoxicillin 375 mg and metronidazole 250 mg, 1 tablet each, every 8 h for 7 days	The antibiotics group showed a higher mean reduction in PD at 12 weeks; however, this difference did not reach statistical significance. Neither treatment resulted in significant improvements in BoP.	Unclear
Shibli et al (2019)^38^	Brazil	40	58.5 ± 11.1/27.5%	PPD > 5 mm, BOP/SOP, marginal bone loss > 4 mm	Systemic, 400 mg metronidazole and 500 mg amoxicillin, TID, last for 14 days	At 1 year, mean PD, mean CAL and proportions of red complex pathogens did not differ significantly between the two groups.	Unclear
De Waal et al, (2021)^40^	Netherlands	62	60.0 ± 10.4/50%; 53.5 ± 11.2/62.5%	marginal bone loss ≥ 2 mm, BOP, SOP, PD ≥ 5 mm	Systemic metronidazole and amoxicillin	No significant differences were observed between both groups for any of the primary or secondary parameters.	Unclear
Ahmed et al, (2020)^39^	Saudi Arabia	40	51.4 ± 6.7/100%; 50.7 ± 5.9/100%	Type 2 diabetes mellitus, BoP, SoP, PD ≥ 6 mm, CAL ≤ 3	Systemic, single dose metronidazole 400 mg and amoxicillin 500 mg	For PD, antibiotics group showed reduced values at the baseline and 6-months of follow-up in patients with type 2 diabetes mellitus.	Unclear
Blanco et al, (2022)^37^	Spain	32	58.31/56%; 60.74/25%	BOP, PD ≥ 6 mm, marginal bone loss ≥ 3 mm	Systemic, 250 mg metronidazole, 2 capsules TID for 7 days	The use of systemic metronidazole as an adjunct therapy resulted in significant additional improvements in clinical, radiographic, and microbiological parameters after 12 months of follow-up.	Low
Park et al (2021)^33^	South Korea	114	61.1/50.9%	PD ≥ 5 mm, BOP, SOP, peri-implant bone loss	Topical metronidazole-minocycline ointment. Weekly, 3 weeks	The treatment success rate (absence of bleeding or suppuration on probing, and sites showing pocket PPD ≥ 5 mm) was higher in the intervention group.	Unclear

BOP, bleeding on probing; SOP, suppuration on probing; PD, probing depth; CAL, clinical attachment level.

**Table 2:** Statistics of the included studies

**Study**	**Sample Size**	**Antibiotic Type**	**PD (mm)**	**BOP %**	**CAL (mm)**
Polymeri et al (2022)^34^	37	Systemic (Amoxicillin + Metronidazole)	2.28 ± 1.49 vs. 1.47 ± 1.95	No effect [Data in figures]	-
Shibli et al (2019)^38^	40	Systemic (Amoxicillin + Metronidazole)	3.1 ± 1.2 vs. 1.8 ± 0.2	0.41 ± 0.39 vs. 0.47 ± 0.31	2.6 ± 1.5 vs. 1.4 ± 0.8
De Waal et al, (2021)^40^	62	Systemic (Amoxicillin + Metronidazole)	1.67 ± 0.82 vs. 1.40 ± 0.80	-	0.97 ± 0.93 vs. 0.96 ± 1.01
Ahmed et al, (2020)^39^	40	Systemic (Single dose amoxicillin + Metronidazole)	6.6 ± 1.2 to 3.9 ± 1.0 vs. 6.9 ± 1.8 to 4.7 ± 1.1	55.8 ± 23.5 to 15.6 ± 8.6 vs. 46.6 ± 24.3 to 19.8 ± 9.4	-
Blanco et al, (2022)^37^	32	Systemic (Metronidazole)	2.53 (1.37–3.69) vs. 1.02 (0.06–1.99)	-	2.14 (0.97–3.30) vs. 0.53 (-0.33–1.39)
Park et al (2021)^33^	114	Local (Metronidazole-Minocycline ointment)	1.88 ± 1.50 vs. 1.28 ± 1.15a	0.50 ± 0.34 vs. 0.33 ± 0.41	-

BOP: bleeding on probing; PD: probing depth; CAL: clinical attachment level

## Discussion

 This systematic review synthesized evidence on the adjunctive use of antibiotics in the nonsurgical management of peri-implantitis. Based on the limited available evidence, our findings confirm that while mechanical debridement remains the cornerstone of therapy, the addition of antibiotics may yield clinical improvements with no considerable side effects. However, interpreting this evidence is not straightforward and requires a critical analysis of the balance between therapeutic benefits and potential long-term risks, particularly the escalating threat of antimicrobial resistance. Despite high heterogeneity arising from different diagnostic criteria, treatment regimens, and study populations, we found that antibiotic therapy was associated with a greater decrease in PD, and the combination of amoxicillin and metronidazole was the most effective intervention in this regard.

 Traditional treatment of peri-implantitis includes both surgical and nonsurgical approaches, aimed at halting disease progression by reducing bacterial load and inflammation.^41-43^ However, a one-size-fits-all approach to treatment is shown to be largely ineffective.^31^ Instead, optimal management necessitates a comprehensive, personalized strategy guided by patient- and site-level risk stratification, as outlined by recent consensus reports.^17^ Evidence indicates that systemic antibiotics, particularly a combination of amoxicillin and metronidazole, provide substantial clinical improvement in patients with diabetes mellitus.^39^ This superior efficacy is likely attributable to their ability to penetrate deep into peri-implant tissues and eradicate pathogens in sites inaccessible to mechanical debridement alone. However, this finding should not be interpreted as a blanket endorsement, as this efficacy was not evident in other included studies. In addition, the clinical dilemma is that these benefits are often short-term, and the routine use of systemic agents for a localized infection contradicts the core principles of antibiotic stewardship.

 Local antibiotic therapy demonstrates consistent efficacy in reducing BOP and improving the subgingival microbial profile. Evidence suggests that the predominant pathogens include *Porphyromonas gingivalis*, *Tannerella forsythia*, *Treponema denticola*, *Fusobacterium nucleatum*, and unique species such as *Filifactor alocis* and *Fretibacterium fastidiosum*, and less common species such as *Staphylococcus* and *Enterobacteriaceae, *which are associated with increased inflammatory response and disease progression.^44^ While their effect on PD reduction is less pronounced than that of systemic agents, they represent a significantly safer therapeutic option with minimal systemic exposure and a lower risk of adverse events.^33^ Their role is therefore critical as a first-line adjunctive therapy for mild-to-moderate peri-implantitis or in patients with contraindications to systemic therapy.

 The clinical community must therefore shift from a reactive to a proactive, risk-based approach. The central challenge highlighted by this review is the paradox of antibiotic use in peri-implantitis. The findings of this review do not support the indiscriminate use of systemic antibiotics. Instead, they suggest a highly selective application, reserving systemic therapy for severe, refractory cases after other treatment modalities have failed. In alignment with modern clinical guidelines, a prudent strategy would involve: 1) initial nonsurgical mechanical debridement; 2) adjunctive use of local antibiotics for mild-to-moderate cases that do not resolve; 3) consideration of systemic antibiotics only for severe cases (e.g., deep pockets, persistent suppuration) or immunocompromised patients, ideally guided by microbiological testing. This stratified approach ensures that the most potent therapies are reserved for when they are most needed, preserving their efficacy for the future.

 The evidence synthesized in this review is constrained by the methodological diversity across the included studies, including variations in treatment protocols, follow-up durations, and case definitions, which complicate the ability to draw definitive conclusions. To address these limitations, future research must adopt a more standardized, robust approach. Another significant gap in the current evidence is the lack of research on the cost-effectiveness of various treatment protocols and the evaluation of patient-reported outcomes.^45,46^ Long-term, high-quality RCTs with standardized protocols are needed to determine the sustainability of treatment outcomes and assess the real-world impact on antibiotic resistance. In addition to optimizing antibiotic use, raising awareness and preventing dental infections are key practical steps that should not be neglected in the dental profession.^47^

 Future research should also focus on cost-effectiveness analyses and patient-reported outcomes to provide a more holistic understanding of the value of different antibiotic strategies. Incorporating these factors into future studies will provide a more holistic understanding of the value of different treatments beyond purely clinical metrics. Moreover, more research is needed to determine the efficacy of treatment protocols explicitly tailored to high-risk groups, such as smokers and diabetics, to develop and validate a truly personalized, risk-based approach to care.

## Conclusion

 Based on the synthesized evidence from 2018 to 2025, the adjunctive use of antibiotics may be associated with enhanced outcomes of nonsurgical peri-implantitis management. Systemic antibiotics may provide short-term benefits, but the risks of antimicrobial resistance and the lack of long-term, high-quality evidence remain critical concerns. Conversely, local antibiotics offer a valuable, well-tolerated alternative that effectively reduces BOP and microbial load, making them a suitable option for mild-to-moderate disease or for patients in whom systemic administration is contraindicated. A critical consideration remains the long-term risk of antimicrobial resistance. Therefore, clinicians must adopt a risk-based, individualized approach, restricting systemic antibiotics to high-risk scenarios and ensuring their use is integrated with meticulous mechanical debridement and a consistent long-term maintenance program. Future high-quality, long-term RCTs are imperative to standardize protocols, evaluate cost-effectiveness, and establish the sustainability of these treatment outcomes.

## Competing Interests

 Currently, Adileh Shirmohammadi is the Editor-in-Chief of the Journal of Advanced Periodontology & Implant Dentistry (JAPID), and Masoumeh Faramarzi and Mehrnoosh Sadighi serve as an Editorial Advisory Board for JAPID. The authors declare no other competing interests concerning authorship and/or publication of this article.

## Data Availability

 All data generated or analyzed during this study are included in this published article.

## Ethical Approval

 This study was approved by the Ethics Committee of Tabriz University of Medical Sciences (Ethics Code: IR.TBZMED.DENTISTRY.REC.1403.096).
